# Mindfulness as a Moderator of the Association Between Eating Disorder Cognition and Eating Disorder Behavior Among a Non-clinical Sample of Female College Students: A Role of Ethnicity

**DOI:** 10.3389/fpsyg.2018.00700

**Published:** 2018-06-25

**Authors:** Akihiko Masuda, Rachel D. Marshall, Janet D. Latner

**Affiliations:** Department of Psychology, University of Hawai‘i at Mānoa, Honolulu, HI, United States

**Keywords:** eating disorder cognition, mindfulness, acting with awareness, eating disorder behavior, ethnicity, race

## Abstract

The present cross-sectional study examined whether mindfulness moderated the association between eating disorder cognition and eating disorder behaviors among Asian American, Black American, and White American female college students in the United States. Participants (*N* = 463, age range = 18–25 years) completed self-report measures online. Results revealed that mindfulness moderated the association between eating disorder cognition and eating disorder behavior in the White American group, but not in Asian American or Black American samples. Future research should replicate these differential findings across ethnic groups and investigate the factors that may contribute to this group difference.

## Introduction

In contrast to previous thinking, disordered eating concerns are now known to be pervasive across all ages, genders, ethnic backgrounds, and sexual orientations in many industrialized nations (Grabe and Hyde, 2006; Mitchison et al., 2014; Lipson and Sonneville, 2017). For example, prevalence rates of anorexia nervosa (AN), bulimia nervosa (BN), and binge eating disorder (BED) in Asian American women were found to be comparable to those in White American women (e.g., Nicdao et al., 2007; Marques et al., 2011). A more recent study (Marques et al., 2011) also shows that White American women and Black American women did not differ from each other in the prevalence rates of AN, BN, or BED. Other research has looked at the associations between gender identity and sexual orientation with disordered eating behaviors. In some studies, eating disorder diagnoses and behavioral eating disturbances (e.g., dieting, past-month vomiting, or laxative use) were more prevalent among sexual minorities (i.e., lesbian, bisexual, or unsure) than heterosexuals (e.g., Diemer et al., 2015). In other studies, sexual minority women endorse fewer compensatory behaviors than heterosexual women (e.g., Lipson and Sonneville, 2017).

In this sociocultural context, mindfulness has been of increasing interest in the field of eating disorder treatment because of its salutary effects across a range of behavioral health issues (Wanden-Berghe et al., 2010; Masuda and Hill, 2013; Katterman et al., 2014). To date, evidence regarding the mechanisms of change in mindfulness remains limited (Keng et al., 2011). One potential mechanism is mindfulness as a moderator of the association between risk factors and maladaptive behaviors (Pidgeon et al., 2013; Adams et al., 2014; Levin et al., 2015): For example, under greater mindfulness the positive association between risk factors, such as eating disorder cognition, and eating disorder behaviors (e.g., binge eating, purging, and restricting) are found to be weaker (Masuda et al., 2012).

The examination of mindfulness as a moderator of the link between eating disorder cognition and eating disorder behavior has not been examined in Asian American, Black American, and White American female college students. Filling this gap is important as these groups of women are known to be particularly vulnerable to disordered eating and body image concerns (Striegel-Moore and Bulik, 2007), and because findings from one sociocultural sample are not necessarily generalizable to other sociocultural groups (Sue, 1999; Cheng and Sue, 2014; Hall et al., 2016). Therefore, the present cross-sectional study examines whether mindfulness moderates the association between eating disorder cognition and eating disorder behavior in groups of Asian American, Black American, and White American college students.

### Eating Disorder Cognition

According to cognitive behavioral models of disordered eating concerns (Vitousek and Hollon, 1990; Fairburn, 2008; Haynos et al., 2016), one major risk factor for eating disorder behavior is dysfunctional cognitions specific to eating disorder concerns. Cognitive behavioral models state that individuals with eating disorders endorse rigid beliefs about the importance of weight regulation, strong beliefs in appearance as the basis of self-worth, and inflexible beliefs in self-control as the basis of self-esteem (Vitousek and Hollon, 1990; Mizes et al., 2000). From this perspective, eating disorder cognitions often reflect, but are not limited to: (a) fear of gaining weight, (b) perceived importance of having an ideal weight and shape as a means of being accepted by others, or (c) perceived self-worth related to self-control over diet and weight (Mizes et al., 2000).

Prospective studies show that eating disorder cognitions, such as thin-ideal internalization, thinness expectancies, and shape/weight overvaluation, have been shown to prospectively predict the development of eating disorder behaviors (Culbert et al., 2015; Stice, 2016). Similarly, cross-sectional investigations have shown that eating disorder cognition is positively associated with eating disorder behaviors and symptoms in samples of college men and women (e.g., Masuda et al., 2012; Wendell et al., 2012) and in a female college sample (e.g., Miller et al., 2009). A few studies directly examined the link between eating disorder cognition and eating disorder behavior in ethnically diverse samples of non-clinical female college students (Miller et al., 2009; Miller and Vaillancourt, 2011; Hill et al., 2013; Moore et al., 2014). These studies revealed a positive association between eating disorder cognition and eating disorder behavior (Miller et al., 2009; Hill et al., 2013; Moore et al., 2014). Internalization of the thin ideal and body dissatisfaction were significantly associated with symptoms of BN and other eating disorder pathology among Black American college women (Lester and Petrie, 1998; Rogers-Wood and Petrie, 2010). One study with a sample of Asian American college women found a significant positive association between eating disorder cognition (e.g., body preoccupation) and eating disorder behavior (Phan and Tylka, 2006).

Of these studies, Moore et al. (2014) also directly examined ethnic group differences in eating disorder cognition and eating disorder behavior. Participants were 421 non-clinical female college students, with 35% self-identifying as “White,” 32% as “Black,” and 17% as “Asian.” Their results showed that being “White” was associated with greater eating disorder cognition than being “non-White,” but there was no ethnic group difference in eating disorder behavior (i.e., the combination of restricting, binge eating, and purging). To date, no study has examined the link between eating disorder cognition and eating disorder behavior separately by each ethnic group (e.g., Asian American, Black American, White American), however.

### Mindfulness

Mindfulness, although varying in definition across investigators (Hayes and Wilson, 2003), is an emotion and behavior regulation process that is conceptually relevant to eating disorder concerns, as eating disturbances and mindfulness may be inversely correlated (Kristeller and Wolever, 2011). One popular view of mindfulness conceptualizes it as an adaptive regulation process of becoming aware of the present moment as measured by the Mindful Attention Awareness Scale (MAAS; Brown and Ryan, 2003; Brown et al., 2007; Chambers et al., 2009). In the mindfulness literature, this aspect of mindfulness is often called acting with awareness (Baer, 2006; Baer et al., 2006).

The significance of mindfulness found in the behavioral health literature parallels contemporary cognitive behavioral models of eating disorders and eating disorder treatment (Fairburn, 2008; Haynos et al., 2016). These cognitive behavioral models postulate that a set of emotion regulation processes, in addition to eating disorder cognition, is relevant to psychopathology specific to eating disorders. In these conceptual models, mindfulness (e.g., acting with awareness) is viewed as an adaptive emotional and behavioral regulation process that may attenuate the development and progression of eating disorder pathology (Kristeller and Wolever, 2011).

A growing body of cross-sectional investigations show that greater mindfulness is associated with fewer eating disorder symptoms in a sample of college women and men (Lavender et al., 2009) and in a sample of ethnically diverse college women (Moore et al., 2014). Furthermore, particularly relevant to the present study, mindfulness was found to moderate the positive association between eating disorder cognition and eating disorder behavior in a sample of college women and men (Masuda et al., 2012). With higher levels of mindfulness, the positive association between eating disorder cognition and eating disorder behavior was significantly smaller than the association of the two eating disorder-related variables under lower levels of mindfulness.

### Present Study

Extant evidence shows that mindfulness moderates the positive association between eating disorder cognition and eating disorder behavior (Masuda et al., 2012). However, no studies have investigated mindfulness as a potential moderator of the association between eating disorder cognition and eating disorder behavior directly in Asian American, Black American, and White American women. Given the findings on the salutary effects of mindfulness (Brown et al., 2007; Chambers et al., 2009; Masuda et al., 2012), it would be reasonable to speculate that mindfulness would moderate the link between eating disorder cognition and eating disorder behavior among groups of Asian American, Black American, and White American female college students. However, proponents of diversity psychology have cautioned us not to assume that any given model, including the modulating effect of mindfulness, is universal; a model should be directly examined with the target sample (Cheng and Sue, 2014). As discussed extensively elsewhere, universality assumptions have often been found to be incorrect (Sue, 1999; Hall et al., 2016). As such, the logical next step was to directly investigate whether mindfulness moderates the association between eating disorder cognition and eating disorder behavior in samples of Asian American, Black American, and White American female college students. We did not examine Latina American college students because we expected that we were not able to recruit enough Latina American college students for the present study.

## Materials and Methods

### Participants

Undergraduate women were recruited from a southeastern public university in the United States through an online research recruitment tool managed by the Department of Psychology. The inclusion criteria for study participants were (a) age between 18- and 25-year-old, (b) self-identified ethnic background of Asian American, Black American, and White American, (c) self-reported height and weight for computing body mass index (BMI). Exclusion criteria were set on (a) age (i.e., 17-year-old and younger or 26-year-old or older), (b) ethnic background (those who self-identify none of the three ethnic group categories of the inclusion criteria), and (c) missing data on self-reported height and weight.

### Procedure

Participants who enrolled in the study were asked to complete an anonymous web-based survey. Prior to the survey, information explaining the purpose of the present study and instructions regarding how to complete the survey were presented on a computer screen. Then participants anonymously filled out demographic information and completed the survey measures.

### Measures

The following measures were used to assess eating disorder cognition, eating disorder behavior, and mindfulness.

#### Eating Disorder Cognition

The Mizes Anorectic Cognitions Questionnaire-Revised (MAC-R; Mizes et al., 2000) is a 24-item self-report questionnaire designed to assess distorted cognitions related to all eating disorders. These cognitions are the fear of weight gain (e.g., “When I see someone who is overweight, I worry that I will be like him/her”), the importance of being thin or attractive to be socially accepted (e.g., “My friends will like me, regardless of how much I weigh”), and self-esteem based on controlled eating habits and weight gain (e.g., “When I overeat, it has no effect on whether or not I feel like a strong person”). Each item is scored on a five-point Likert scale, ranging from 1 (strongly disagree) to 5 (strongly agree), with a total score derived from the sum of all responses. Total scores range from 24 to 120 with higher scores indicating greater disordered eating-related dysfunctional cognitions. In a previous study conducted with a non-clinical college sample, Cronbach’s alpha for the MAC-R was 0.89 (Masuda et al., 2010). In the current study, Cronbach’s alphas of this measure were 0.87, 0.90, and 0.85 for Asian American, White American, and Black American groups, respectively.

#### Eating Disorder Behavior

Based on previous findings (Masuda et al., 2012), the sum of nine behavioral items in the 26-item version of Eating Attitudes Test (EAT-26; Garner et al., 1982) was used to measure behavioral symptoms of eating disorders. These items clearly capture the behavioral symptoms of disordered eating (e.g., “I avoid eating when I am hungry”). More specifically, seven items reflect restricting and AN (e.g., “I cut my food into small pieces”), one item reflects purging (i.e., “I vomit after I have eaten”), and one item reflects binge eating (i.e., “I have gone on eating binges when I feel that I may not be able to stop”). All items are scored on a six-point Likert scale: never (0), rarely (0), sometimes (0), often (1), very often (2), or always (3). The total score of the behavioral scale (EAT-26 B) ranges from 0 to 27. A previous study demonstrated an adequate internal consistency of this scale with Cronbach’s alpha of 0.72 (Masuda et al., 2012). In the present study, Cronbach’s alphas of EAT-26 B were 0.67, 0.56, and 0.66 for Asian American, White American, and Black American groups, respectively.

#### Mindfulness

The MAAS (Brown and Ryan, 2003) is a 15-item self-report measure, which is designed to assess the frequency of mindlessness, the opposite of the construct of mindfulness, over time (e.g., “It seems I am running automatic without much awareness of what I’m doing”). Participants rate the degree to which they function mindlessly in daily life, using a six-point Likert scale ranging from 1 (almost always) to 6 (almost never). Total scores range from 15 to 90, with higher scores denoting greater mindfulness. The MAAS has good internal consistency (i.e., Cronbach’s alpha), ranging from 0.82 to 0.87 (Brown and Ryan, 2003). In the current study, Cronbach’s alphas of this measure were 0.86, 0.88, and 0.89 for Asian American, White American, and Black American groups, respectively.

### Data Analysis

In the present set of analyses, we first examined zero-order correlations among study variables that were collected as interval variables for each ethnic group. For sexual orientation that was nominally categorized (dummy coded as 0 = heterosexual, 1 = sexual minority), we conducted a series of one-way analyses of variance (ANOVAs) to examine its main effect on other study variables separately for each ethnic group. Subsequently, we conducted a multivariate analysis of variance (MANOVA) to investigate the main effects of ethnicity and sexual orientation as well as the ethnicity × sexual orientation interaction effect on eating disorder behavior (EAT-26 B), eating disorder cognition (MAC-R), mindfulness (MAAS), age, and BMI.

**FIGURE 1:**
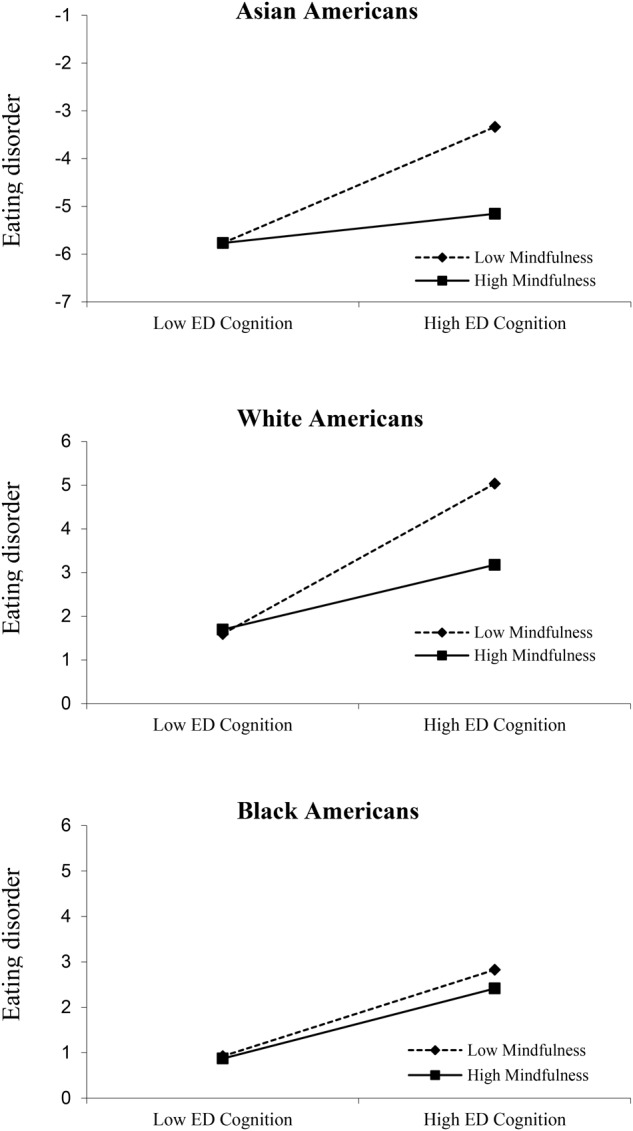
Interaction between MAC-R scores and MAAS scores. MAAS scores were split at +1 SD above the mean and –1 SD below the mean. EAT-26-B, Eating Attitudes Test-26 item; MAC-R, Mizes Anorectic Cognition Questionnaire-Revised; MAAS, Mindfulness Attention Awareness Scale.

We then ran a hierarchical multiple regression for each ethnic group to investigate whether eating disorder cognition and mindfulness accounted for unique variances in eating disorder behavior and whether mindfulness moderated the association between disordered eating cognition and disordered eating behavior. The first step included age, sexual orientation, and BMI as covariates. The second step included eating disorder cognition and mindfulness. The two-way interactions of eating disorder cognition × mindfulness were entered in the third step. To probe the specific form of the interaction, the association between eating disorder cognition and eating disorder behavior was computed at high (+1 SD) and low (–1 SD) levels of mindfulness (**Figure 1**).

## Results

Six-hundred forty-six undergraduate women were recruited and completed the present web-based survey. Of those, 112 participants were excluded from the present study because they self-identified as either Latina (*n* = 60), Pacific Islander (*n* = 1), other (*n* = 11), or bicultural (*n* = 40). An additional 29 individuals were removed from the study because they failed to self-report their weight and height for computing their BMI. Finally, 42 participants were removed because they were either 17-year-old or younger or older than 25-year-old. The final sample consisted of 463 undergraduates ranging in age from 18 to 25 years old (mean age = 19.31, *SD* = 1.61 years), including 112 Asian American, 108 Non-Hispanic White American, and 243 Black American women. This ethnic distribution was representative of the university from which the present study participants were recruited. Sexual minorities were represented in each ethnic group, with seven Asian American, 14 White American, and 25 Black American students reporting sexual minority status (i.e., “bisexual” or “homosexual”).

### Associations Among Study Variables by Ethnic Group

Descriptive statistics and correlations among the study variables are shown in **Tables 1, 2**. In the Asian American group, mindfulness (MAAS) was negatively associated with eating disorder cognition (MAC-R) and eating disorder behavior (EAT-26-B). Eating disorder cognition was positively associated with eating disorder behavior and BMI, and BMI was positively associated with eating disorder behavior. Additionally, being a sexual minority was associated with greater BMI in the Asian American group, *F*(1,110) = 4.36, *p* < 0.05.

**Table 1:** Means and standard deviations of study variables by sexual orientation in each ethnic group.

	Asian American			White American		
Means (standard deviations)	Heterosexual (*n* = 105)	Sexual minority (*n* = 7)	Total (*n* = 112)	Heterosexual (*n* = 94)	Sexual minority (*n* = 14)	Total (*n* = 108)
Eating disorder behavior (EAT-26 B)	2.24 (3.10)	4.43 (5.50)	2.38 (3.31)	2.02 (2.64)	2.21 (2.64)	2.05 (2.62)
Eating disorder cognition (MAC-R)	63.81 (13.81)	57.14 (18.18)	63.39 (14.12)	64.31 (15.60)	68.79 (18.72)	64.90 (16.01)
Mindfulness (MAAS)	55.21 (10.30)	54.86 (4.49)	55.19 (10.02)	52.00 (11.04)	43.71 (11.28)	50.92 (11.37)
Age	19.18 (1.34)	19.57 (2.07)	19.21 (1.38)	19.60 (1.83)	20.57 (2.47)	19.72 (1.94)
BMI	21.62 (3.29)	24.55 (7.01)	21.80 (3.65)	22.95 (4.17)	20.86 (3.58)	22.68 (4.14)

	**Black American**			**Overall**		
**Means (standard deviations)**	**Heterosexual (*n* = 218)**	**Sexual Minority (*n* = 25)**	**Total** **(*n* = 243)**	**Heterosexual (*n* = 417)**	**Sexual Minority (*n* = 46)**	**Total** **(*n* = 463)**

Eating disorder behavior (EAT-26 B)	1.66 (2.61)	1.84 (2.13)	1.67 (2.56)	1.88 (2.75)	2.34 (3.05)	1.93 (2.78)
Eating disorder cognition (MAC-R)	59.91 (14.27)	64.60 (12.54)	60.40 (14.15)	61.88 (14.58)	64.74 (15.60)	62.17 (14.69)
Mindfulness (MAAS)	57.61 (12.97)	51.24 (10.03)	56.95 (12.83)	55.74 (12.11)	49.50 (10.47)	55.12 (12.09)
Age	19.14 (1.45)	19.56 (2.04)	19.18 (1.52)	19.25 (1.52)	19.87 (2.19)	19.31 (1.61)
BMI	24.92 (5.15)	26.50 (7.53)	25.08 (5.44)	23.65 (4.74)	24.49 (6.84)	23.73 (4.98)

*N = 463; EAT-26, Eating Attitudes Test-26 item; B, behavior; MAC-R, Mizes Anorectic Cognition Questionnaire-Revised; MAAS, Mindfulness Attention Awareness Scale; BMI, body mass index.*

**Table 2:** Zero-order relations between all variables.

	1	2	3	4	5
**Asian American (*n* = 112)**					
1. Eating disorder behavior (EAT-26 B)	–				
2. Eating disorder cognition (MAC-R)	0.29 **	–			
3. Mindfulness (MAAS)	–0.22 *	–0.27 **	–		
4. Age	0.03	–0.07	–0.01	–	
5. BMI	0.24 *	0.35 **	–0.08	0.02	–
**White American (*n* = 108)**					
1. Eating disorder behavior (EAT-26 B)	–				
2. Eating disorder cognition (MAC-R)	0.55 **	–			
3. Mindfulness (MAAS)	–0.28 **	–0.32 **	–		
4. Age	0.06	0.03	–0.14	–	
5. BMI	0.16	0.38 **	–0.04	0.06	–
**Black American (*n* = 243)**					
1. Eating disorder behavior (EAT-26 B)	–				
2. Eating disorder cognition (MAC-R)	0.35 **	–			
3. Mindfulness (MAAS)	–0.12	–0.23 **	–		
4. Age	–0.01	–0.00	0.10	–	
5. BMI	0.11	0.29 **	0.05	0.10	–

*^∗^*p* < 0.05, ^∗∗^*p* < 0.01; EAT-26, Eating Attitudes Test-26 item; B, behavior; MAC-R, Mizes Anorectic Cognition Questionnaire-Revised; MAAS, Mindfulness Attention Awareness Scale; BMI, body mass index; different superscripts across means indicate pairwise significant differences in means, ^a^indicating the difference between Asian American and White American groups; ^b^indicating the difference between Asian American and White American group, ^c^indicating the difference between the White American and Black American groups.*

In the White American group, mindfulness was negatively associated with eating disorder cognition and with eating disorder behavior. Eating disorder cognition was positively associated with BMI. BMI was not significantly related to eating disorder behavior or mindfulness. Being a sexual minority was associated with lower mindfulness, *F*(1,106) = 6.83, *p* < 0.05.

In the Black American group, mindfulness was inversely associated with eating disorder cognition, but not with eating disorder behavior. Eating disorder cognition was positively associated with eating disorder behavior and BMI. BMI was not associated with eating disorder behavior. Being a sexual minority was associated with lower mindfulness, *F*(1,241) = 5.63, *p* < 0.05.

### Role of Ethnicity and Sexual Orientations on Study Variables

Significant effects for ethnic group, sexual orientation, and their interaction were followed by pairwise comparisons with a Bonferroni correction to maintain the overall alpha at 0.05. Results revealed main effects of ethnicity and sexual orientation, as well as a significant ethnicity × sexual orientation interaction (*Fs* > 2.14. *p* < 0.05). More specifically, we found a main effect of ethnicity on all predicted variables (*Fs* > 3.35, *p* < 0.05), except eating disorder cognition. Similarly, we found a main effect of sexual orientation on mindfulness and age (*F* > 4.53, *p* < 0.05). Furthermore, the MANOVA results revealed a significant ethnicity × sexual orientation interaction effect on BMI (*F* = 3.20. *p* < 0.05).

Pairwise comparisons revealed the Asian American group endorsed significantly more eating disorder behavior than the Black American group (*p* = 0.03). No other ethnic group difference was found in eating disorder behavior. Asian American and Black American groups reported greater mindfulness than the White American groups (*ps* < 0.04). There was no difference in mindfulness between the Asian American and Black American groups. In BMI, the Black American group showed greater BMI than the Asian American and White American groups (*ps* < 0.05). The Asian American and White American groups did not differ in BMI. Finally, the White American group was found to be older than the Black American group (*p* < 0.03). No other ethnic group differences were found in age.

Regarding the main effect of sexual orientation, the sexual minority group was found to be less mindful and older than the heterosexual group (*ps* < 0.04). Finally, with respect to the ethnicity × sexual orientation interaction on BMI, the sexual minority group reported higher BMI than the heterosexual group among Asian Americans (*p* < 0.04). There was a trend of sexual orientation effect on BMI in the White American group (*p* < 0.08). Being a sexual minority was related to lower BMI than being heterosexual in the White American group.

### Associations of Eating Disorder Cognition and Mindfulness to Disordered Eating Behavior by Ethnic Group

**Table 3** presents the final step of a hierarchical linear regression that examined the associations of eating disorder cognition and mindfulness with eating disorder behavior by ethnic group. In the Asian American group, after adjusting for the effects of age, sexual orientation, and BMI, eating disorder cognition (β = 0.23, *p* < 0.05), but not mindfulness (β = -0.15, *p* > 0.05), was found to be a unique predictor of eating disorder behavior (Step 2; *R^2^*Δ = 0.09, *p* < 0.01). The final step of the regression analysis revealed eating disorder cognition as a unique predictor of eating disorder behavior (β = 0.22, *p* < 0.05). The final step also showed a trend toward a two-way interaction between mindfulness and eating disorder cognition (β = -0.16, *p* = 0.09; Step 3 *R^2^*Δ = 0.03, *p* = 0.09). As shown in **Figure 1**, there is a trend of high mindfulness attenuating the strength of positive association between eating disorder cognition and eating disorder behavior (simple slope: *t* = 1.835, *p* = 0.069).

**Table 3:** Final step of a hierarchical linear regression examining the role of eating disorder cognition and mindfulness on eating disorder behavior.

Eating disorder behaviors (EAT-26-B)	*b*	*SE*	β	*p*
**Asian American**				
*Intercept*	–2.90	4.53		0.532
*Direct effects*				
Age	0.14	0.22	0.06	0.519
BMI	0.10	0.09	0.11	0.246
Sexual orientation	2.20	1.26	0.16	0.084
Disordered eating cognitions (MAC-R)	0.79 *	0.35	0.22	0.027
Mindfulness (MAAS)	–0.50	0.37	–0.13	0.181
*Moderating effects*				
MAAS × MAC-R	–0.59	0.34	–0.16	0.089
**White American**				
*Intercept*	2.52	2.52		0.473
*Direct effects*				
Age	0.04	0.11	0.03	0.742
BMI	–0.04	0.06	–0.06	0.513
Sexual orientation	–0.77	0.67	–0.10	0.254
Disordered eating cognitions (MAC-R)	0.96 **	0.28	0.39	0.001
Mindfulness (MAAS)	–0.38	0.25	–0.14	0.125
*Moderating effects*				
MAAS × MAC-R	–0.49 *	0.22	–0.23	0.028
**Black American**				
*Intercept*	1.88	2.09		0.410
*Direct effects*				
Age	–0.00	0.10	–0.00	0.976
BMI	0.01	0.03	0.01	0.853
Sexual orientation	–0.17	0.52	–0.02	0.748
Disordered eating cognitions (MAC-R)	0.92 **	0.18	0.34	0.000
Mindfulness (MAAS)	–0.12	0.15	–0.05	0.427
*Moderating effects*				
MAAS × MAC-R	–0.09	0.15	–0.04	0.554

*^∗^*p* < 0.05, ^∗∗^*p* < 0.01; EAT-26, Eating Attitudes Test-26 item version; BMI, body mass index; MAC-R, Mizes Anorectic Cognition Questionnaire-Revised; MAAS, Mindfulness Attention Awareness Scale.*

In the White American group, after adjusting for the effects of age, sexual orientation, and BMI, eating disorder cognition (β = 0.54, *p* < 0.05), but not mindfulness (β = -0.13, *p* > 0.05), was a unique predictor of eating disorder behavior (Step 2 *R^2^*Δ = 0.29, *p* < 0.01). The final step of the regression analysis revealed a significant two-way interaction between mindfulness and eating disorder cognition (β = -0.23, *p* < 0.05; Step 3 *R^2^*Δ = 0.03, *p* < 0.05). In particular, high mindfulness attenuated the strength of positive association between eating disorder cognition and eating disorder behavior, as shown in **Figure 1** (simple slope: *t* = 4.327, *p* < 0.01).

In the Black American group, after adjusting for the effects of age, sexual orientation, and BMI, eating disorder cognition (β = 0.34, *p* < 0.01), but not mindfulness (β = -0.05, *p* > 0.05), was a unique predictor of eating disorder behavior (Step 2; *R^2^*Δ = 0.11, *p* < 0.01). The final step of the regression analysis revealed that the interaction was not significant, and that eating disorder cognition was the only unique predictor (β = 0.34, *p* < 0.01) after the two-way interaction of mindfulness and eating disorder cognition was entered into the model (Step 3 *R^2^*Δ = 0.00, *p* > 0.05; see **Figure 1**).

## Discussion

The present study examined mindfulness as a potential moderator of the association between eating disorder cognition and eating disorder behavior. An ethnically diverse, non-clinical sample included groups of Asian American, White American, and Black American women. In all three groups, bivariate correlations showed that eating disorder cognition was significantly correlated with eating disorder behavior. Among White Americans and Asian Americans, both cognition and behavior were also associated with mindfulness, consistent with past results (Moore et al., 2014). However, among Black Americans, mindfulness was associated with only eating disorder cognition, but not with eating disorder behavior. These novel results suggest that the association between eating disordered behavior and mindfulness found previously in other samples (Masuda et al., 2012; Moore et al., 2014) may not function in the same way among Black American women. These findings emphasize the importance of examining associations independently in different ethnic groups, rather than assuming generalizability or universality across cultures (Sue, 1999; Hall et al., 2016; Schaefer et al., 2018).

Hierarchical regression also examined the unique association of eating disorder cognition, mindfulness, and their interaction with eating disorder behavior, after adjusting for BMI, age, and sexual orientation. As with the bivariate analyses, different results emerged across ethnic groups. Among White American women, mindfulness moderated the association between eating disorder cognition and eating disorder behavior. Consistent with past research (Masuda et al., 2012), this finding suggests the expression of eating disturbances is moderated by the level of mindfulness: the positive association between eating disorder cognition and eating disorder behavior is weaker under greater mindfulness. However, among Asian Americans, only a trend toward the interaction emerged. Importantly, the interaction was not significant among Black Americans. Again, this pattern of results highlights the importance of investigating theories within distinct cultural groups, as the associations between putative maintenance variables can differ, with potentially different treatment implications.

It is possible that the slightly lower levels of eating disorder cognition and behavior among Black American women might have been related to the absence of their interaction in this group. It is also possible that the higher BMI among the Black American group suggests that the behavioral eating disturbances in this group might have taken a slightly different form than those in the other two groups, and perhaps were more focused on concerns about overeating or loss-of-control eating rather than dietary restraint (Wildes et al., 2001; Schaefer et al., 2018).

These findings have important theoretical implications. In all three groups, eating disorder cognition was associated with eating disorder behavior, consistent with cognitive behavioral theories of the maintenance of eating disorders (Vitousek and Hollon, 1990; Fairburn, 2008). In addition, the associations between mindfulness and both eating disorder cognition (across all groups) and eating disorder behavior (among Asian and White Americans) support more recent theoretical models of psychopathology emphasizing the importance of mindfulness as an adaptive emotional and behavioral process that may regulate and mitigate the development of eating disorder symptomatology (Keng et al., 2011; Kristeller and Wolever, 2011; Haynos et al., 2016).

Both mindfulness-based treatments and cognitive behavioral treatments aim to improve self-awareness and self-regulation surrounding eating behavior in order to regain control over eating, food choices, and responses to both emotional (e.g., distress) and physical (e.g., hunger and satiety) cues. The treatments might use different methods to achieve this goal (e.g., self-monitoring and meal planning in cognitive behavioral therapy, mindfulness meditation and appetite tracking in mindfulness-based approaches), but enhancing conscious control over eating is a key treatment goal across treatment modalities. The present study further emphasizes an important role for mindfulness in the treatment and prevention of disordered eating problems. Acceptance- and mindfulness-based treatments have shown preliminary efficacy in reducing disordered eating problems (Kristeller and Wolever, 2011; Masuda and Hill, 2013). However, most participants in these studies have been individuals from non-minority groups.

The present investigation has several limitations. The current data were collected from students attending an urban university in the southeastern United States. As such, generalizability to other ethnic groups and other geographic areas is unknown. Further research is also needed to investigate the associations examined here among women from other ethnic groups (e.g., Latina Americans), older women, and among individuals of higher BMI. The scales used in the present study have not been fully psychometrically tested and validated across diverse ethnic groups, although they have been used in prior research with similar populations (Masuda et al., 2012, 2017; Moore et al., 2014).

The coefficient alphas of the measures used here were all in the adequate range, except those of EAT-26 B. The somewhat lower coefficient alphas of EAT-26 B in across the three ethnic groups suggest that the construct of eating disorder behavior that was used in a previous study with a sample of ethnically diverse college women (Moore et al., 2014) may not be adequately applicable to specific ethnic groups of college women. Furthermore, the construct of eating disorder behavior used in the present study predominantly reflects restricting more so than binge eating/purging. As such, future studies should examine the association of eating disorder cognition and mindfulness to specific types of eating disorder behaviors systematically.

As with all correlational research, the present study does not allow for causal conclusions to be drawn, and longitudinal and experimental studies are warranted. Finally, the construct of mindfulness examined in the present study focused on the awareness of present moment experience, and did not include other domains of mindfulness highlighted by other studies, such as acceptance and non-judgment (Kabat-Zinn, 2003; Bishop et al., 2004; Baer et al., 2006). Thus, it is possible that different patterns of results would emerge if other measures of mindfulness were used.

Despite these limitations, the present study extends our understanding of the associations between eating disorder cognition, eating disorder behavior, and mindfulness, and the attenuating role of mindfulness in the association between disordered cognition and behavior. Importantly, this study examined these associations in samples of previously understudied populations, Asian Americans and Black Americans, as well as White Americans. The present results serve as a reminder of the importance of investigating hypothesized associations across different populations and avoiding assumptions of generalizability. The results also underscore the importance of further research devoted to better understanding the maintenance factors of eating-related psychopathology across cultures, especially among Black American and Asian American women (Rogers-Wood and Petrie, 2010; Cheng et al., 2017). It is possible that additional variables not yet examined in the context of eating disorders but identified as important in other domains (e.g., interdependence, spirituality, discrimination; Hall et al., 2016) might play a role in the development and maintenance of eating disorders across cultures, and further research is strongly encouraged.

## Ethics Statement

This study was carried out in accordance with the recommendations of the institutional and/or national research committee. The protocol was approved by the institutional research committee. All subjects gave written informed consent in accordance with the Declaration of Helsinki.

## Data Availability Statement

The datasets for this study can be found at the open science framework.

## Author Contributions

AM designed and executed the study, assisted with data analysis, and wrote the paper. RM collaborated in the writing and editing of the final manuscript. JL collaborated on the design and wrote the paper.

## Conflict of Interest Statement

The authors declare that the research was conducted in the absence of any commercial or financial relationships that could be construed as a potential conflict of interest. The reviewer HE and handling Editor declared their shared affiliation.

## References

[B1] Adams C. E. McVay M. A. Stewart D. W. Vinci C. Kinsaul J. Benitez L. (2014). Mindfulness ameliorates the relationship between weight concerns and smoking behavior in female smokers: a cross-sectional investigation. *Mindfulness* 5 179–185. 10.1007/s12671-012-0163-9 24778746 PMC3999963

[B2] Baer R. A. (2006). *Mindfulness-Based Treatment Approaches: Clinician’s Guide to Evidence Base and Applications.* San Diego, CA: Elsevier Academic Press.

[B3] Baer R. A. Smith G. T. Hopkins J. Krietemeyer J. Toney L. (2006). Using self-report assessment methods to explore facets of mindfulness. *Assessment* 13 27–45. 10.1177/1073191105283504 16443717

[B4] Bishop S. R. Lau M. Shapiro S. Carlson L. Anderson N. D. Carmody J. (2004). Mindfulness: a proposed operational definition. *Clin. Psychol. Sci. Pract.* 11 230–241. 10.1093/clipsy.bph077

[B5] Brown K. W. Ryan R. M. (2003). The benefits of being present: mindfulness and its role in psychological well-being. *J. Pers. Soc. Psychol.* 84 822–848. 10.1037/0022-3514.84.4.822 12703651

[B6] Brown K. W. Ryan R. M. Creswell J. D. (2007). Mindfulness: theoretical foundations and evidence for its salutary effects. *Psychol. Inq.* 18 211–237. 10.1080/10478400701598298

[B7] Chambers R. Gullone E. Allen N. B. (2009). Mindful emotion regulation: an integrative review. *Clin. Psychol. Rev.* 29 560–572. 10.1016/j.cpr.2009.06.005 19632752

[B8] Cheng H. L. Tran A. G. Miyake E. R. Kim H. Y. (2017). Disordered eating among Asian American college women: a racially expanded model of objectification theory. *J. Couns. Psychol.* 64 179–191. 10.1037/cou0000195 28277732

[B9] Cheng J. K. Y. Sue S. (2014). “Addressing cultural and ethnic minority issues in the acceptance and mindfulness movement,” in *Mindfulness and Acceptance in Multicultural Competency: A Contextual Approach to Sociocultural Diversity in Theory and Practice* eds Masuda A. Masuda A. (Oakland, CA: Context Press/New Harbinger Publications) 21–37.

[B10] Culbert K. M. Racine S. E. Klump K. L. (2015). Research review: what we have learned about the causes of eating disorders–a synthesis of sociocultural, psychological, and biological research. *J. Child Psychol. Psychiatry* 56 1141–1164. 10.1111/jcpp.12441 26095891

[B11] Diemer E. W. Grant J. D. Munn-Chernoff M. A. Patterson D. A. Duncan A. E. (2015). Gender identity, sexual orientation, and eating-related pathology in a national sample of college students. *J. Adolesc. Health* 57 144–149. 10.1016/j.jadohealth.2015.03.003 25937471 PMC4545276

[B12] Fairburn C. G. (2008). *Cognitive Behavior Therapy and Eating Disorders.* New York, NY: Guilford Press.

[B13] Garner D. M. Olmsted M. P. Bohr Y. Garfinkel P. E. (1982). The eating attitudes test: psychometric features and clinical correlates. *Psychol. Med.* 12 871–878. 10.1017/s0033291700049163 6961471

[B14] Grabe S. Hyde J. S. (2006). Ethnicity and body dissatisfaction among women in the United States: a meta-analysis. *Psychol. Bull.* 132 622–640. 10.1037/0033-2909.132.4.622 16822170

[B15] Hall G. C. Yip T. Zárate M. A. (2016). On becoming multicultural in a monocultural research world: a conceptual approach to studying ethnocultural diversity. *Am. Psychol.* 71 40–51. 10.1037/a0039734 26766764

[B16] Hayes S. C. Wilson K. G. (2003). Mindfulness: method and process. *Clin. Psychol. Sci. Pract.* 10 161–165. 10.1093/clipsy/bpg018

[B17] Haynos A. F. Forman E. M. Butryn M. L. Lillis J. (2016). *Mindfulness and Acceptance in for Treating Eating Disorders and Weight Concerns: Evidence-Based Interventions.* Oakland, CA: Context Press/New Harbinger Publications.

[B18] Hill M. L. Masuda A. Latzman R. D. (2013). Body image flexibility as a protective factor against disordered eating behavior for women with lower body mass index. *Eat. Behav.* 14 336–341. 10.1016/j.eatbeh.2013.06.003 23910777

[B19] Kabat-Zinn J. (2003). Mindfulness-based interventions in context: past, present, and future. *Clin. Psychol. Sci. Pract.* 10 144–156. 10.1093/clipsy/bpg016

[B20] Katterman S. N. Kleinman B. M. Hood M. M. Nackers L. M. Corsica J. A. (2014). Mindfulness meditation as an intervention for binge eating, emotional eating, and weight loss: a systematic review. *Eat. Behav.* 15 197–204. 10.1016/j.eatbeh.2014.01.005 24854804

[B21] Keng S.-L. Smoski M. J. Robins C. J. (2011). Effects of mindfulness on psychological health: a review of empirical studies. *Clin. Psychol. Rev.* 31 1041–1056. 10.1016/j.cpr.2011.04.006 21802619 PMC3679190

[B22] Kristeller J. L. Wolever R. Q. (2011). Mindfulness-based eating awareness training for treating binge eating disorder: the conceptual foundation. *Eat. Disord.* 19 49–61. 10.1080/10640266.2011.533605 21181579

[B23] Lavender J. M. Jardin B. F. Anderson D. A. (2009). Bulimic symptoms in undergraduate men and women: contributions of mindfulness and thought suppression. *Eat. Behav.* 10 228–231. 10.1016/j.eatbeh.2009.07.002 19778752

[B24] Lester R. Petrie T. A. (1998). Physical, psychological, and societal correlates of bulimic symptomatology among African American college women. *J. Couns. Psychol.* 45 315–321. 10.1037/0022-0167.45.3.315

[B25] Levin M. E. Luoma J. B. Haeger J. A. (2015). Decoupling as a mechanism of change in mindfulness and acceptance. *Behav. Modif.* 39 870–911. 10.1177/0145445515603707 26349756

[B26] Lipson S. K. Sonneville K. R. (2017). Eating disorder symptoms among undergraduate and graduate students at 12 U.S. colleges and universities. *Eat. Behav.* 24(Suppl. C) 81–88. 10.1016/j.eatbeh.2016.12.003 28040637

[B27] Marques L. Alegria M. Becker A. E. Chen C. N. Fang A. Chosak A. (2011). Comparative prevalence, correlates of impairment, and service utilization for eating disorders across US ethnic groups: implications for reducing ethnic disparities in health care access for eating disorders. *Int. J. Eat. Disord.* 44 412–420. 10.1002/eat.20787 20665700 PMC3011052

[B28] Masuda A. Goodnight B. L. Ng S. Y. Ward Schaefer L. Tully E. C. Chan W. Y. (2017). Help-seeking stigma in Asian American college women: the role of disordered eating cognitions and psychological inflexibility. *Int. J. Adv. Couns.* 39 188–201. 10.1007/s10447-017-9291-1

[B29] Masuda A. Hill M. L. (2013). Mindfulness as therapy for disordered eating: a systematic review. *Neuropsychiatry* 3 433–447. 10.2217/npy.13.36 24854804

[B30] Masuda A. Price M. Anderson P. L. Wendell J. W. (2010). Disordered eating-related cognition and psychological flexibility as predictors of psychological health among college students. *Behav. Modif.* 34 3–15. 10.1177/0145445509351569 20051522

[B31] Masuda A. Price M. Latzman R. D. (2012). Mindfulness moderates the relationship between disordered eating cognitions and disordered eating behaviors in a non-clinical college sample. *J. Psychopathol. Behav. Assess.* 34 107–115. 10.1007/s10862-011-9252-7 22888181 PMC3415312

[B32] Miller J. L. Vaillancourt T. (2011). “Rethinking the eating disorder continuum: a categorical approach to abnormal eating,” in *Handbook of Behavior, Food and Nutrition* eds Preedy V. R. Watson R. R. Martin C. R. (New York, NY: Springer) 1411–1429.

[B33] Miller J. L. Vaillancourt T. Hanna S. E. (2009). The measurement of ‘eating-disorder-thoughts’ and ‘eating-disorder-behaviors’: implications for assessment and detection of eating disorders in epidemiological studies. *Eat. Behav.* 10 89–96. 10.1016/j.eatbeh.2009.02.002 19447350

[B34] Mitchison D. Hay P. Slewa-Younan S. Mond J. (2014). The changing demographic profile of eating disorder behaviors in the community. *BMC Public Health* 14:943. 10.1186/1471-2458-14-943 25213544 PMC4246495

[B35] Mizes J. S. Christiano B. Madison J. Post G. Seime R. Varnado P. (2000). Development of the mizes anorectic cognitions questionnaire—revised: psychometric properties and factor structure in a large sample of eating disorder patients. *Int. J. Eat. Disord.* 28 415–421. 10.1002/1098-108X(200012)28:4<415::AID-EAT9>3.0.CO;2-Z 11054788

[B36] Moore M. Masuda A. Hill M. L. Goodnight B. L. (2014). Body image flexibility moderates the association between disordered eating cognition and disordered eating behavior in a non-clinical sample of women: a cross-sectional investigation. *Eat. Behav.* 15 664–669. 10.1016/j.eatbeh.2014.08.021 25289446

[B37] Nicdao E. G. Hong S. Takeuchi D. T. (2007). Prevalence and correlates of eating disorders among Asian Americans: results from the national Latino and Asian American study. *Int. J. Eat. Disord.* 40 S22–S26. 10.1002/eat.20450 17879986

[B38] Phan T. Tylka T. L. (2006). Exploring a model and moderators of disordered eating with Asian American college women. *J. Couns. Psychol.* 53 36–47. 10.1037/0022-0167.53.1.36

[B39] Pidgeon A. Lacota K. Champion J. (2013). The moderating effects of mindfulness on psychological distress and emotional eating behaviour. *Aust. Psychol.* 48 262–269. 10.1111/j.1742-9544.2012.00091.x

[B40] Rogers-Wood N. A. Petrie T. A. (2010). Body dissatisfaction, ethnic identity, and disordered eating among African American women. *J. Couns. Psychol.* 57 141–153. 10.1037/a0018922 21133566

[B41] Schaefer L. M. Burke N. L. Calogero R. M. Menzel J. E. Krawczyk R. Thompson J. K. (2018). Self-objectification, body shame, and disordered eating: testing a core mediational model of objectification theory among White, Black, and Hispanic women. *Body Image* 24 5–12. 10.1016/j.bodyim.2017.10.005 29172061 PMC5869145

[B42] Stice E. (2016). Interactive and mediational etiologic models of eating disorder onset: evidence from prospective studies. *Annu. Rev. Clin. Psychol.* 12 359–381. 10.1146/annurev-clinpsy-021815-093317 26651521

[B43] Striegel-Moore R. H. Bulik C. M. (2007). Risk factors for eating disorders. *Am. Psychol.* 62 181–198. 10.1037/0003-066x.62.3.181 17469897

[B44] Sue S. (1999). Science, ethnicity, and bias: Where have we gone wrong? *Am. Psychol.* 54 1070–1077. 10.1037/0003-066x.54.12.1070 15332528

[B45] Vitousek K. Hollon S. D. (1990). The investigation of schematic content and processing in eating disorders. *Cogn. Ther. Res.* 14 191–214. 10.1007/BF01176209

[B46] Wanden-Berghe R. G. Sanz-Valero J. Wanden-Berghe C. (2010). The application of mindfulness to eating disorders treatment: a systematic review. *Eat. Disord.* 19 34–48. 10.1080/10640266.2011.533604 21181578

[B47] Wendell J. W. Masuda A. Le J. K. (2012). The role of body image flexibility in the relationship between disordered eating cognitions and disordered eating symptoms among non-clinical college students. *Eat. Behav.* 13 240–245. 10.1016/j.eatbeh.2012.03.006 22664403

[B48] Wildes J. E. Emery R. E. Simons A. D. (2001). The roles of ethnicity and culture in the development of eating disturbance and body dissatisfaction: a meta-analytic review. *Clin. Psychol. Rev.* 21 521–551. 10.1016/S0272-7358(99)00071-9 11413866

